# Straight outta clusters: differing gene duplication patterns for alkaloid biosynthesis and floral traits in California poppy

**DOI:** 10.1093/plcell/koag051

**Published:** 2026-02-28

**Authors:** Linhan Sun

**Affiliations:** Assistant Features Editor, the Plant Cell, American Society of Plant Biologists; Department of Plant Pathology and Environmental Microbiology, The Pennsylvania State University, University Park, PA 16802, United States

When California is referred to as the “Golden State,” most people think of the Gold Rush, the Golden Gate Bridge, or the Golden State Warriors. Plant enthusiasts, however, may instead think of its state flower—California poppy (*Eschscholzia californica*)—and its characteristic golden-orange blossoms. Aside from its vibrant coloration, California poppy is also touted as a rich source of benzylisoquinoline alkaloids (BIAs; Fig. 1a), a class of plant specialized metabolites that hold both deep cultural significance and substantial pharmaceutical value (Anderson 2007; Becker et al. 2023).

**Figure 1:**
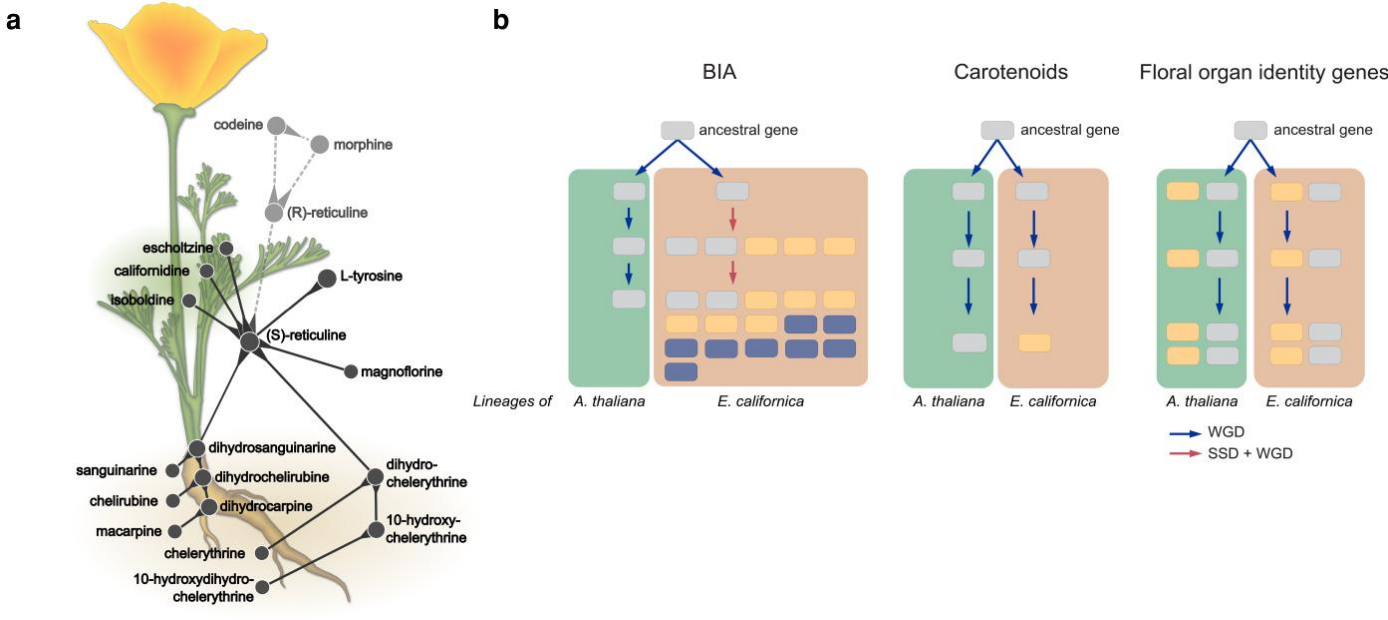
**a)** Localization of major BIAs in California poppy (*eschscholzia californica*). **b)** Patterns of gene duplication and expression divergence of BIA, carotenoid, and floral organ identity-associated gene families in California poppy. WGD, whole genome duplication; SSD, small-scale duplication. Adapted from Rössner et al. (2025), Fig. 1B and Fig. 6.

In new work in *The Plant Cell*, **Le-Han Rössner, Clemens Rössner, and colleagues (Rössner et al. 2025)** took a step further in deciphering the secrets of California poppy by reporting its first haplotype-resolved genome. This genome allowed them to perform comparative genomics analyses of genes involved in 3 biological pathways that make California poppy so special: BIA biosynthesis, petal pigmentation (carotenoid biosynthesis; Barrell et al. 2010), and flower development (regulated by homeotic MADS-box genes).

The authors first examined gene families involved in BIA biosynthesis in California poppy and other species in the same order of Ranunculales. For comparison, BIA biosynthesis genes were also examined in selected core eudicots, rice (monocot), and *Amborella*. Ranunculales species harbor substantially more hierarchical orthogroups (HOGs) for the BIA biosynthesis pathway than the other angiosperm species examined. This enrichment suggests that many BIA-related gene families are of Ranunculales-specific origin. Within BIA-related gene families, HOGs that encode enzymes at branching points or end points of the BIA biosynthesis pathway contain more members than the HOGs for core-pathway enzymes. Notably, gene duplications in BIA-associated gene families are largely species-specific across Ranunculales, indicating independent diversification of BIA metabolism in different taxa.

A long-standing paradigm in plant-specialized metabolite biosynthesis research is that genes encoding enzymes for distinct steps in the same pathway occasionally cluster together in the genome, forming biosynthetic gene clusters (BGCs; Nützmann and Osbourn 2014). BIA biosynthesis in California poppy is unique in this regard, as most of the BIA-related gene clusters are composed of members within a single gene family. By leveraging an expression atlas of California poppy generated in this study, the authors further examined expression patterns of BIA genes, especially the physically clustered paralogs. Many of BIA genes show tissue- or stage-specific expression, which could contribute to distinct tissue-specific BIA profiles (Fig. 1a). More importantly, clustered BIA genes within the same family tend to coexpress, suggesting that divergence in expression patterns has delayed the expansion of gene families.

In contrast to BIA biosynthesis genes, genes involved in carotenoid metabolism and flower development tell a different story: HOGs related to these two pathways contain much fewer members than BIA-associated HOGs, and these HOGs usually lack lineage specificity. These genes also do not form clusters like BIA genes. Comparative expression analyses between California poppy and Arabidopsis further showed markedly higher transcript accumulation of conserved carotenoid genes in reproductive tissues of California poppy, likely contributing to its bright orange petals. In contrast, homeotic MADS-box genes regulating floral identity exhibit a high degree of conservation in expression between California poppy and Arabidopsis.

The California poppy genome and transcriptome highlighted how gene duplication can create evolutionary novelty through distinct trajectories, as illustrated by the 3 most notable features of this plant (Fig. 1b): massive expansion of coexpressed, clustered BIA biosynthetic genes; comparatively stable carotenoid gene numbers with shifted expression in reproductive tissues; and conserved floral identity genes in both gene numbers and expression. Here, California poppy provides a “golden” example of gene duplication as a strong driving force that shapes the “endless forms most beautiful” in plant diversity.

## Recent related articles in *The Plant Cell*:

Laforest et al. (2025) reported the genome and transcriptome of *Mitragyna parvifolia* (Rubiaceae), with a particular emphasis on gene evolution contributing to another type of alkaloids, monoterpene indole alkaloids (MIAs), in this family. In contrast to the largely single-family clusters of BIA genes in California poppy, MIA biosynthetic genes in Rubiaceae are organized into more canonical, multi-enzyme BGCs.

Rao et al. (2024) investigated the role of Arabidopsis Nudix hydrolase 23 (NUDX23) in post-translational regulation of carotenoid biosynthesis, the same pigment underlying the iconic orange petal coloration of California poppy.

Zhao et al. (2023) examined how gene duplication and function diversification of flower developmental genes, including homeotic MADS-box genes, have shaped flower morphology in Delphinieae, a tribe within the same order (Ranunculales) with California poppy.

## Data Availability

None to declare.

## Dive Curated Terms

The following phenotypic, genotypic, and functional terms are of significance to the work described in this paper:

BIA Gramene: chebi: 22750
BIA Araport: chebi: 22750
